# Potential for re-emergence of wheat stem rust in the United Kingdom

**DOI:** 10.1038/s42003-018-0013-y

**Published:** 2018-02-08

**Authors:** Clare M. Lewis, Antoine Persoons, Daniel P. Bebber, Rose N. Kigathi, Jens Maintz, Kim Findlay, Vanessa Bueno-Sancho, Pilar Corredor-Moreno, Sophie A. Harrington, Ngonidzashe Kangara, Anna Berlin, Richard García, Silvia E. Germán, Alena Hanzalová, David P. Hodson, Mogens S. Hovmøller, Julio Huerta-Espino, Muhammed Imtiaz, Javed Iqbal Mirza, Annemarie F. Justesen, Rients E. Niks, Ali Omrani, Mehran Patpour, Zacharias A. Pretorius, Ramin Roohparvar, Hanan Sela, Ravi P. Singh, Brian Steffenson, Botma Visser, Paul M. Fenwick, Jane Thomas, Brande B. H. Wulff, Diane G. O. Saunders

**Affiliations:** 1 John Innes Centre, Norwich Research Park, Norwich, NR4 7UH UK; 2 University of Exeter, Exeter, EX4 4QD UK; 3 Pwani University, 195-80108 Kilifi, Kenya; 4 Department of Forest Mycology and Plant Pathology, Swedish University of Agricultural Sciences, Uppsala, 750 07 Sweden; 5 Instituto Nacional de Investigación Agropecuaria (INIA) La Estanzuela, Mailbox 39173 Colonia, Uruguay; 6 Crop Research Institute, Ruzyně, 161 06 Praha 6 Czech Republic; 7 International Maize and Wheat Improvement Center (CIMMYT), 5689 Addis Ababa, Ethiopia; 8 Aarhus University, Flakkebjerg, 4200 Denmark; 9 Campo Experimental Valle de México INIFAP, Texcoco, C. P. 56237 Mexico; 10 CIMMYT-Pakistan, Islamabad, 44000 Pakistan; 11 Crop Disease Research Program, National Agriculture Research Center, Islamabad, 44000 Pakistan; 12 Wageningen University, Wageningen, 6700 The Netherlands; 13 Faculty of Agriculture, Department of Plant Breeding and Biotechnology, University of Tabriz, Tabriz, 5166616471 Iran; 14 University of the Free State, Bloemfontein, 9301 South Africa; 15 Seed and Plant Improvement Institute, Agricultural Research, Education and Extension Organization (AREEO), 4119 Karaj, Iran; 16 Tel Aviv University, Tel Aviv, 69978 Israel; 17 CIMMYT, Apdo. Postal 6-641, D. F. México, 06600 Mexico; 18 University of Minnesota, St. Paul, 55455 MN USA; 19 Limagrain UK Ltd, Woolpit, IP30 9UP UK; 20 National Institute of Agricultural Botany, Cambridge, CB3 0LE UK

## Abstract

Wheat stem rust, a devastating disease of wheat and barley caused by the fungal pathogen *Puccinia graminis* f. sp. *tritici*, was largely eradicated in Western Europe during the mid-to-late twentieth century. However, isolated outbreaks have occurred in recent years. Here we investigate whether a lack of resistance in modern European varieties, increased presence of its alternate host barberry and changes in climatic conditions could be facilitating its resurgence. We report the first wheat stem rust occurrence in the United Kingdom in nearly 60 years, with only 20% of UK wheat varieties resistant to this strain. Climate changes over the past 25 years also suggest increasingly conducive conditions for infection. Furthermore, we document the first occurrence in decades of *P. graminis* on barberry in the UK . Our data illustrate that wheat stem rust does occur in the UK and, when climatic conditions are conducive, could severely harm wheat and barley production.

## Abstract (web summary)

Clare Lewis et al. report the first identification in nearly 60 years of a cultivated wheat plant infected with the fungal pathogen *Puccinia graminis* f.sp. *tritici* (wheat stem rust) in the United Kingdom. They find that only 20% of UK wheat varieties are resistant to this strain and urge growers to resume resistance breeding programs.

## Introduction

Wheat stem rust, caused by the fungal pathogen *Puccinia graminis* f. sp. *tritici*, has recently re-emerged in Europe. In 2013, Germany experienced its first major outbreak in decades after an unusually cold spring was followed by high early summer temperatures^1^. In addition, both bread and durum wheat were ravaged by stem rust in Sicily in 2016, marking the largest European outbreak for many years^2^. Stem rust is a long-standing threat to wheat and barley production. A cornerstone of the Green Revolution in the mid-to-late twentieth century was breeding for resistance against stem rust^3^. However, new supervirulent wheat stem rust isolates such as the notorious Ug99 race group have emerged in Africa and their impending spread poses a significant threat to global food security^4^. In addition, as climate conditions shift, the earlier-maturing wheat varieties that were once bred to avoid inoculum build-up^5^ could be at risk, as evidenced by recent reports of stem rust outbreaks in Europe.

Beyond breeding for resistance, large-scale removal of the alternate host barberry (*Berberis* spp.)^6^ reduced the potential for enhancing the pathogen’s genetic diversity and the spawning of new races, e.g., radically reducing the number of *P. graminis* f. sp. *tritici* races in the United States from 17 to 8 per year after eradication^3^. Over the past decade, however, barberry planting has been reinitiated and is increasing rapidly in many major wheat-growing regions^3^. The presence of common barberry has the potential not only to enhance the pathogen’s genetic diversity but also to provide a seasonal bridge for stem rust in temperate zones^7^. Dormant stem rust spores may overwinter and germinate in the spring to infect the alternate host barberry, providing inoculum to re-infect primary grass and cereal hosts. Barberry eradication in the United Kingdom during the late nineteenth and early twentieth century was a massive success, breaking the disease cycle and driving wheat stem rust to almost complete extinction^8^, with the last recorded epidemic in the United Kingdom in 1955^9^. Accordingly, overwintering has been perceived as unlikely in Europe for decades due to the absence of both *P. graminis* f. sp. *tritici* and the alternate host in most areas. However, in 2017 Sweden reported the first occurrence of a sexual population of wheat stem rust that was derived from barberry signifying a worrying turn for wheat stem rust in Europe^10^.

Here we report the first record of wheat stem rust in the United Kingdom in nearly 60 years, and that only 20% of UK wheat varieties are resistant to this strain. We also identified for the first time in many decades a stem rust fungus on its alternate host common barberry in the United Kingdom, where it was identified within meters of a barley field. Our results indicate that, with alterations in climatic conditions over the past 25 years, suggesting increasingly conducive conditions for fungal pathogen growth and infection, wheat stem rust is becoming an increasing threat to European wheat and barley production.

## Results

### UK-01 belongs to the epidemic race ‘Digalu’

**Fig. 1:**
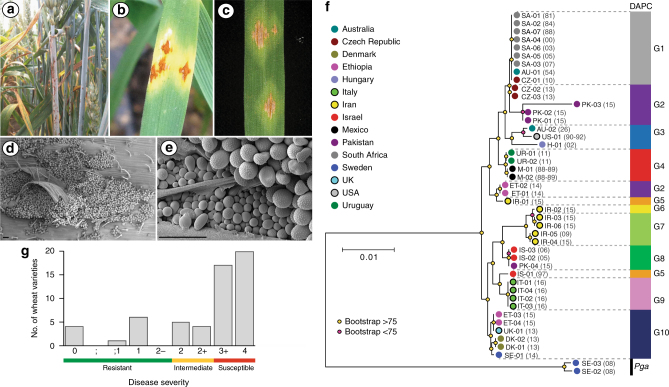
First recorded occurrence of wheat stem rust in the United Kingdom for 58 years. **a** Images of leaf and stem infection of a single wheat plant in the United Kingdom in 2013 with characteristic *P. graminis* f. sp. *tritici* uredinia. **b**, **c** Distinctive *P. graminis* f. sp. *tritici* diamond-shaped uredinia on wheat leaves induced by the UK-01 *P. graminis* f. sp. *tritici* isolate. **d, e** Scanning electron micrographs of erumpent pustules with typical spiny oval urediniospores. Bars represent 50 μm. **f** Phylogenetic analysis was carried out with the 2013 UK-01 isolate and a further forty-two *P. graminis* f. sp. *tritici* isolates from fourteen countries, with two *P. graminis* f. sp. *avenae* isolates as outliers. We used the third codon position of 16,482 gene models (7,348,046 sites) and a maximum-likelihood model for the phylogenetic analysis. Multivariate analysis with discriminant analyses of principal components (DAPC) using 306,960 biallelic synonymous single-nucleotide polymorphism (SNP) sites assigned the isolates to 10 genetic groups. Scale bar represents nucleotide substitutions per site; parenthesis contains year of isolation; *Pga*, *P. graminis* f. sp. *avenae*. **g** Stem rust reaction assays of 57 wheat varieties including the UK recommended list that were infected with UK-01 indicated that only 20% were resistant to infection. On the 0–4 scale, infection types of 0, ;, ;1, 1, and 2 – were considered as representing an incompatible interaction, 2 and 2+ were considered intermediate, and 3+ and 4 represented a compatible interaction between the host genotype and pathogen (*X* axis)

In 2013, we found a single wheat plant in southern England infected with stem rust. This UK isolate, which we named UK-01, induced characteristic *P. graminis* f. sp. *tritici* uredinia on wheat, which were erumpent, diamond-shaped, and full of spiny oval urediniospores on the stem and leaves (Fig. 1a–e). To compare UK-01 with global stem rust populations, we carried out comparative population genetic analysis using 42 *P. graminis* f. sp. *tritici* isolates from fourteen countries and two *P. graminis* f. sp. *avenae* isolates as outliers (Supplementary Table 1). First, we undertook either full-genome or transcriptome sequencing on all isolates, including UK-01. High-quality reads were aligned to the *P. graminis* f. sp. *tritici* reference genome^11^ and phylogenetic analysis undertaken using 7,348,046 sites and a maximum-likelihood approach (Fig. 1f and Supplementary Data 1). To evaluate genetic subdivisions within this population, we used 306,960 synonymous single-nucleotide polymorphism (SNP) sites and discriminant analysis of principal components to define genetic groups (Supplementary Fig. 1), which assigned the isolates to 10 groups of homogeneous individuals (Fig. 1f and Supplementary Fig. 2).

**Fig. 2:**
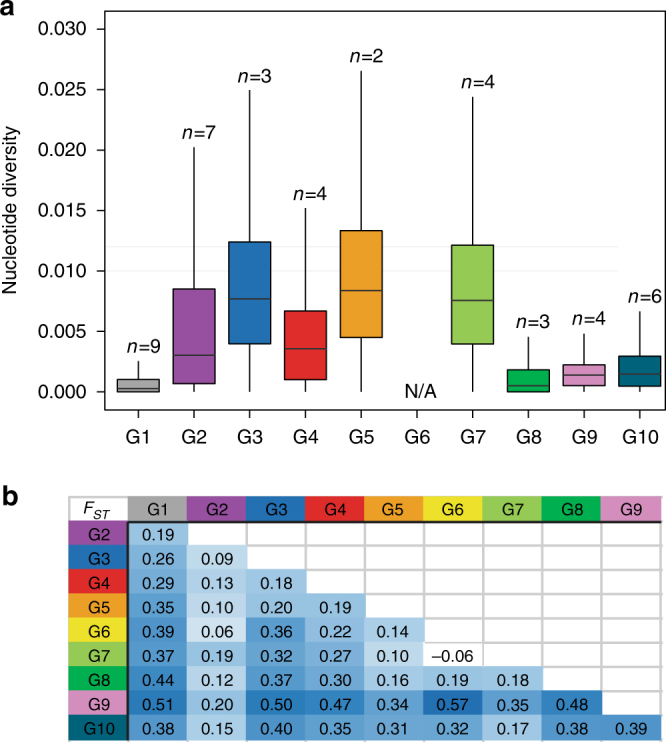
Within and between population diversity for the ten genetic groups of *P. graminis* f. sp. *tritici* isolates. **a** Genetic Group 10 (G10), which contained UK-01, was among the groups with the lowest level of nucleotide diversity. The number (*n*) of isolates per group is indicated. Group 6 had only one isolate and therefore calculation of the nucleotide diversity was not applicable (N/A). Box plot excludes outliers. **b** Genetic Group 9 (G9), which contained isolates from the recent stem rust outbreak in Sicily, was the most distantly related to all other genetic groups (*F*_ST_ 0.20–0.57). Pair-wise comparisons between the ten genetic groups of *P. graminis* f. sp. *tritici* were calculated using Wright’s *F*_ST_ statistic

Notably, UK-01 was most closely related to *P. graminis* f. sp. *tritici* isolates from Ethiopia collected in 2014 and 2015, and Danish and Swedish isolates detected in single locations in 2013 and 2014, respectively, with all aforementioned isolates clustering in a single genetic group with little diversity (Fig. 1f and Fig. 2; median nucleotide diversity 1.46 × 10^3^). The collection of the Ethiopian isolates in 2014–15 succeeded a severe stem rust epidemic facilitated by the widespread planting of a single bread wheat variety. ‘Digalu’ was planted on ~ 30% of the wheat acreage and then succumbed to stem rust infection in late 2013, leading to rapid, wide-scale production losses^12^. Originally detected in Turkey, the ‘Digalu’-infecting race, TKTTF, has spread across the Middle East^12^ and recently into Europe, where it was the dominant race in the 2013 German outbreak^1^. The close genetic proximity between the UK isolate and the Ethiopian, Danish, and Swedish TKTTF-like variants^13^ suggests that UK-01 belongs to the TKTTF (or a closely related) race. This relationship was further supported through virulence profiling, where UK-01 was inoculated onto a series of differential wheat varieties known as the North American Wheat Stem Rust Differential set and disease severity recorded in seedling tests 14–16 days post inoculation. This analysis showed that UK-01 behaved identically to the TKTTF race (Table 1). We speculate that the TKTTF race likely spread across Europe from south to north via wind-borne urediniospore dispersal along the west European track^14^ from a common source in 2013.

**Table 1:** Virulence profiling of *P. graminis* f. sp. *tritici* isolate UK-01 on the international differential set of wheat varieties

*P. graminis* isolate UK-01 was screened for its virulence phenotype across the North American Wheat Stem Rust Differential set. Five plants for each of the 20 lines were tested, with two independent biological replicates. Disease severity was assessed on the first seedling leaf using the United States Department of Agriculture scoring system^41^, where 0, ;, ;1, 1, and 2– were considered as representing an incompatible interaction, 2 and 2+ were considered intermediate, and 3+ and 4 represented a compatible interaction between the host genotype and pathogen

Lewis et al. report the first identification in nearly 60 years of a cultivated wheat plant infected with the fungal pathogen *P. graminis* f.sp. *tritici* (wheat stem rust) in the United Kingdom. They find that only 20% of UK wheat varieties are resistant to this strain and urge growers to resume resistance breeding programs

### UK-01 may infect over 80% of current UK wheat varieties

To explore the potential threat stem rust poses to UK wheat production, we assessed the susceptibility of current UK wheat varieties to UK-01. We inoculated UK-01 onto seedlings of 43 wheat varieties from the UK Recommended List^15^ and 14 older varieties that are still grown on a small scale. Of these 57 varieties, 37 showed a high degree of susceptibility in seedling tests, 9 displayed an intermediate reaction, and 11 were resistant to some degree to infection (Fig. 1g and Supplementary Table 2). Thus, only 20% of wheat varieties currently grown in the United Kingdom are estimated to be resistant to the stem rust isolate UK-01.

### Identification of *P. graminis* inoculum on barberry

**Fig. 3:**
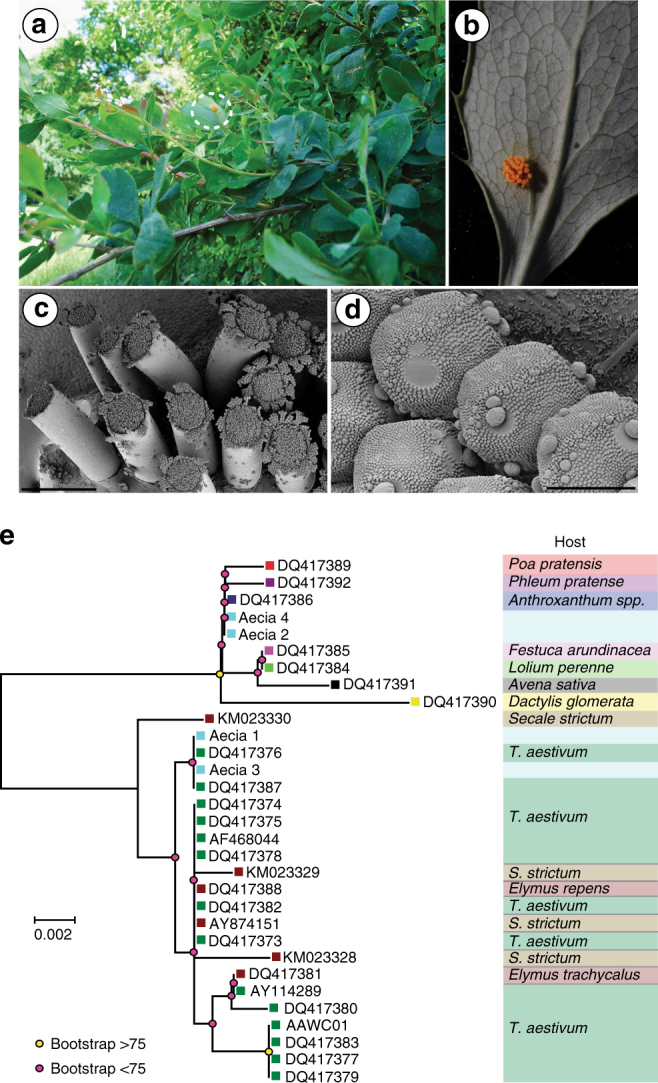
Identification of stem rust in the United Kingdom on the alternate host *B. vulgaris*. **a**, **b** Images of aecia on the abaxial side of *B. vulgaris* leaves typical of cluster cup rust infection. **c**, **d** Scanning electron micrographs of tube-like aecial structures on *B. vulgaris*. Bars represent 50 μm. **e** Phylogenetic analysis of the internal transcribed spacer (ITS) region amplified from four aecia (Aecia 1–4) identified in a single location in the UK, with 27 isolates of different *P. graminis formae speciales* using a Neighbor-joining method. Scale bar indicates nucleotide substitutions per site; names are accession numbers from NCBI

In the United Kingdom, replanting of the alternate host of stem rust, common barberry (*Berberis vulgaris*), is keenly advancing, particularly due to a habitat conservation programme for the endangered barberry carpet moth *Pareulype berberata*^16^ (Supplementary Fig. 3). To examine the potential hazard represented by barberry as a source of inoculum, we examined bushes in three locations in the east of England in June 2017. At one location, we identified a hedgerow that was intermixed with *B. vulgaris* within a meter of a barley field (Supplementary Fig. 4). We found numerous yellow, tube-like aecial structures on the abaxial side of leaves (Fig. 3a–d), which are typical of cluster cup rust of barberry caused by *P. graminis*^17^. Genotypic characterization of the internal transcribed spacer (ITS) region from four aecia confirmed the identification of *P. graminis* (Genbank MF684370-3). Subsequent phylogenetic analysis grouped two aecial sequences in a clade with *P. graminis* f. sp. *tritici* from wheat*, P. graminis* f. sp. *secalis* from wild rye (*Secale strictum* subsp. *africanum*), and *P. graminis* from couch grass (*Elymus* spp.), which are too similar to distinguish using classical gene sequence analysis^18^, but are all capable of infecting barley and, to differing degrees, wheat^19,20^. The other two sequences were more closely related to *P. graminis* from wild grasses (Fig. 3e).

To evaluate the ability of selected aecia to cause disease on wheat and barley, we carried out controlled infection assays with the resulting aeciospores on one barley and two wheat varieties. None of the selected aecia induced symptoms on the two selected wheat varieties. However, spores from 5 of 9 aecia tested were able to infect the selected barley variety (Supplementary Fig. 4c), thereby suggesting a potential threat to the adjacent barley crop. If confirmed as *P. graminis* f. sp. *tritici*, this would be of particular concern as large-scale screening of barley germplasm over the years has only identified seven resistance loci^21^, most of which have been overcome^22^. Further evidence is needed to establish the risk to barley of any UK-derived stem rust isolates. However, this does constitute the first evidence for many decades that the stem rust fungus is overwintering in the UK and able to infect its alternate host common barberry in the spring.

The planting of thousands of common barberry plants across the United Kingdom continues to accelerate (Supplementary Fig. 3) and each medium-sized barberry bush is capable of producing over 20,000 seeds that can remain dormant for up to 10 years^3,23^. Thus, the bushes will be increasingly available to harbor rust pathogens that utilize barberry as a sexual host. Indeed, following the repeal of the barberry exclusion law in Sweden, the oat stem rust fungus for which common barberry is an alternate host has substantially increased in genetic diversity^24^. Furthermore, Sweden recently reported the first occurrence of a sexual population of wheat stem rust derived from barberry for the first time in decades^10^. In the United Kingdom, the gravest concern regards the well-established wheat yellow rust pathogen, *Puccinia striiformis* f. sp. *tritici*, which is closely related to *P. graminis* f. sp. *tritici*. Although not currently known to undergo sexual reproduction in Europe, the unusually high quantities of teliospores produced by recent emergent *P. striiformis* races^25^ could potentially expedite infection as common barberry becomes increasingly prevalent.

### Increasing climatic risk of stem rust re-emergence in the United Kingdom

**Fig. 4:**
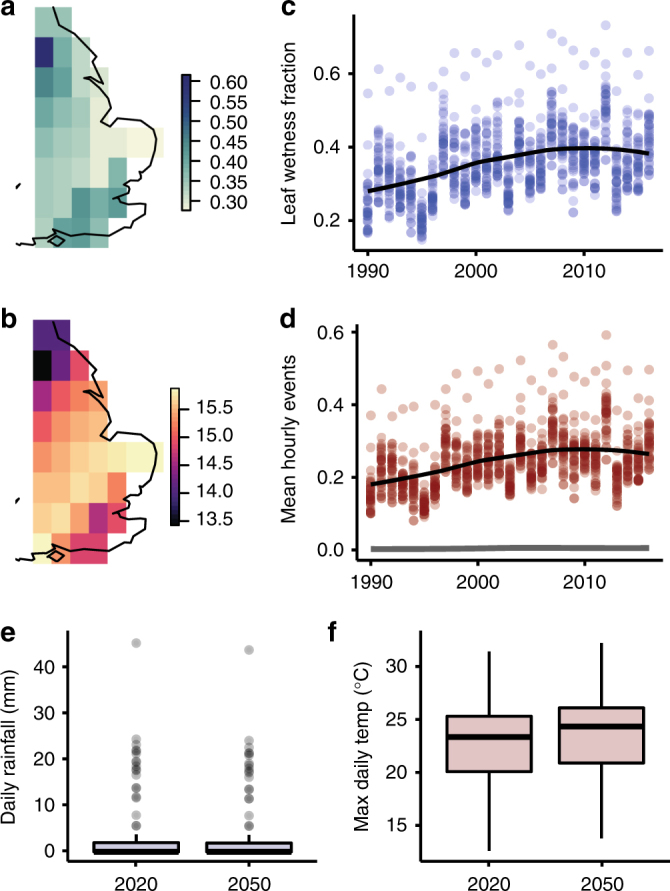
Weather-driven risk model indicates increasingly conducive conditions for fungal pathogen growth over the past 25 years. **a** Mean leaf wetness fraction, 1990–2016, Japanese 55-year Reanalysis (JRA55). Values are the fraction of hours during the summer when canopy surface moisture was above zero. **b** Mean canopy temperature, 1990–2016, JRA55. Values are canopy temperature during summer months when the JRA55 canopy moisture fraction was above zero. **c** Annual leaf wetness fraction, 1990–2016. Each point is the mean per pixel. The curve is a loess fit. **d** Annual mean hourly appressorium formations, 1990–2016. Each point is the mean per pixel, with the model assuming a new cohort of spores deposited in each hour. The black curve is a loess fit. The grey curve is the loess fit to the modeled mean hourly leaf penetrations, values of which were approximately one tenth of the appressorium formations. **e** Daily rainfall distributions, 2020 vs. 2050. Values represent summer days from 99 runs of the MarkSim daily weather generator for 17 CMIP GCMs, for a point near Cambridge. **f** Daily maximum temperature, 2020 vs. 2050. Values derived as in **e**

To determine whether alterations in climatic conditions could further enhance the risk of wheat stem rust in the United Kingdom, we developed a probabilistic model for spore germination rates, appressorium formation and penetration rates over the past quarter century, and drove the model using microclimate estimates from the JRA-55 climate re-analysis^26^ (Supplementary Fig. 5). These growth stages of the fungus require liquid moisture on the leaf surface. The warm temperatures and high light levels required for stem rust penetration^27^ suggest that the disease is most likely to occur in the summer; therefore, we focused on weather data for June–August from 1990 to 2016. The estimated canopy liquid surface water was above zero 30–40% of the time during the summer months, a value that was slightly greater in the far south and northwest of the wheat-growing region (Fig. 4a). The warmest temperatures during the wet periods were found in the central parts of the wheat growing region (Fig. 4b). The fraction of time the canopy was wet increased significantly from 1990 to 2016, suggesting increasingly conducive conditions for fungal pathogen growth and infection (Fig. 4c). The modelled spore germination and appressorium formation rates were strongly determined by leaf wetness, as the optimal temperature range for these processes is wide (see Methods). The predicted rates of penetration, which is dependent on higher temperatures and light levels, as well as on leaf moisture, were an order of magnitude lower than predicted appressorium formation rates (Fig. 4d)^27^. Overall, the model for germination and appressorium formation during wet periods from 1990 to 2016 indicated a trend of increasing risk to 2006, levelling off in the past few years with the exception of the very wet year in 2012 (Fig. 4d).

Next, we considered climate change projections for 2050 that predict very slight drying (Fig. 4e) and slight warming (Fig. 4f) of the central part of the wheat-growing area in the United Kingdom^28^. This analysis suggested that although the risk of spore germination and appressorium formation may increase, the wet conditions required for leaf penetration are unlikely to become more common in the mid-term. However, the high levels of sexual recombination possible via barberry infection could enhance the likelihood of emergence of *P. graminis* f. sp. *tritici* variants that are adapted to prevailing conditions. Worryingly, the Ug99 race has already been reported in preliminary analysis to have a higher level of aggressiveness at cooler temperatures compared to other wheat stem rust races^29^. This ability to adapt could facilitate proliferation into new geographic regions in a similar manner to the high temperature-tolerant races of *P. striiformis* f. sp. *tritici*^30^.

## Conclusions

The Nobel laureate Norman Borlaug foresaw that “the greatest ally of the pathogen is our short memory”^3^. We recommend the re-initiation of resistance breeding and a review of the mass plantation of common barberry to preclude re-planting near arable land and thereby limit the ability of the pathogen to rapidly overcome any introduced resistance and/or climatic constraints to safeguard European cereals from a large-scale re-emergence of wheat stem rust.

## Methods

### RNA-seq of *P. graminis*-infected leaf samples

A total of 12 *P. graminis*-infected wheat leaf samples were collected and stored to maintain nucleic acid integrity in RNAlater solution (Thermo Fisher Scientific, Paisley, UK; Supplementary Table 1). Samples were subsequently subjected to RNA extraction using a Qiagen RNeasy Mini Kit (Qiagen, UK) and the quality and quantity of extracted RNA assessed using an Agilent 2100 Bioanalyzer (Agilent Technologies, UK). Complementary DNA libraries were prepared using the Illumina TruSeq RNA Sample Preparation Kit (Illumina, USA) and sequenced on the Illumina HiSeq 2500 machine at the Earlham Institute, UK. Adapter and barcode trimming and quality filtering were performed on the 97–101 bp paired-end reads using the FASTX-Toolkit (version 0.0.13.2). Reads were then aligned to the *P. graminis* assembly^11^ and SNP calling undertaken using SAMtools (version 0.1.19)^31^, considering only sites with a minimum depth of coverage of 20 ×. Allelic frequencies were determined for each SNP site and those ranging from 0.2 to 0.8 were classified as heterokaryotic sites and those with other frequencies classified as homokaryotic sites. SNP sites that induced synonymous and non-synonymous substitutions were identified using SnpEff, version 3.6^32^.

### Genome sequencing of *P. graminis* isolates

Genomic DNA was extracted from dried urediniospores of 31 *P. graminis* isolates (Supplementary Table 1) using the cetyl trimethyl ammonium bromide (CTAB) method^33^. The gDNA libraries were prepared using the Illumina TruSeq DNA Sample preparation Kit (Illumina) and library quality confirmed before sequencing using the Agilent 2100 Bioanalyzer (Agilent Technologies). Libraries were sequenced on the Illumina HiSeq 2500 machine at the Earlham Institute or Novogene, China. We also included in our analysis publicly available genome sequence data from two *P. graminis* isolates collected in Australia^34^. Following data filtering, the 76–150 bp pair-end reads for each sample were independently aligned to the *P. graminis* assembly^11^ and SNP calling performed as described above but with a minimum threshold of 10 × depth of coverage.

### Phylogenetic analysis of *P. graminis* isolates

Phylogenetic analyses of *P. graminis* isolates were performed using gene sequences to avoid over-representation of isolates subjected to full genome sequencing (compared with those used for transcriptome sequencing) and a maximum likelihood approach. First, nucleotide sites that differed from the reference genome were identified and recorded if they had a minimum of 10 × depth of coverage for gDNA samples and 20 × depth of coverage for RNA-seq samples. Next, sites that were identical to the reference were recorded if they satisfied a minimum of 2 × coverage. Using these data, synthetic gene sets were generated that incorporated these sites for each isolate using the method described previously^35^. The third codon position of 16,482 genes was used to generate maximum likelihood trees using RaxML 8.0.20 with 100 replicates using the rapid bootstrap algorithm^36^. Phylogenetic trees were visualized in MEGA 7.0^37^.

### Population genetic analysis

The existence of population subdivisions was investigated using nonparametric multivariate clustering. This method allows the clustering of isolates without a priori knowledge (e.g., geographical locations or date of collection) that prevents different genetic lineages being grouped together when identified in the same region and thereby interfering with the detection of admixture events^38^. To reduce any potential bias of selection, only sites that introduced a synonymous change in at least one isolate were listed and the nucleotide at this position extracted for all isolates. Multivariate analyses were performed using discriminant analyses of principal components (DAPCs) implemented in the Adegenet package in the R environment^39^, which is a non-parametric approach used without any predetermined genetic model. The number of population clusters (Kmax) were identified using the Bayesian information criterion (BIC), as suggested^39^.

Next, the synthetic gene sets per isolate that were generated for the phylogenetic analysis were combined in one file for all isolates within a genetic group following the grouping identified using DAPC. Nucleotide diversity was then determined using these gene sequences and DnaSP, version 5.10.1^40^ for each genetic group. The diversity within each genetic group was calculated using the statistic *Pi* divided by the number of analysed sites (only sites with < 5% of missing data were included: max_missing_freq = 0.05). To determine the proportion of total genetic variance attributable to inter-population polymorphisms, the synthetic gene sets of all isolates were combined in one file and the Weir and Cockeram *F*_ST_ (egglib statistics: WCst) calculated pairwise for all population pairs with max_missing_freq = 0.05. For both calculations, the number of analyzed sites and mutations was determined using the Egglib statistics lseff and S, respectively.

### Virulence profiling of *P. graminis* isolates

First, *P. graminis* isolate UK-01 was screened for its virulence phenotype across the North American Wheat Stem Rust Differential set, which includes the host resistance genes *Sr5*, *Sr21*, *Sr9e*, *Sr7b* (set 1),* Sr11*,* Sr6*,* Sr8a*,* Sr9g* (set 2), *Sr36*,* Sr9b*,* Sr30*,* Sr17* (set 3), *Sr9a*,* Sr9d*,* Sr10*,* SrTmp* (set 4), and *Sr24*, *Sr31*, *Sr38*, and *SrMcN* (set 5). Five plants for each of the 20 lines were tested under controlled environmental conditions, with two independent biological replicates. Spores were distributed onto test plants in a mixture with talcum powder and plants were incubated for 48 h in polythene bags containing a small amount of water at 18 °C (8 h night) and 24 °C (16 h day), before being removed and grown for a further 14 days. Infection types were assessed on the first seedling leaf using the United States Department of Agriculture scoring system^41^, where 0, ;, ;1, 1, and 2– were considered as representing an incompatible interaction, 2 and 2+ were considered intermediate and 3+ and 4 represented a compatible interaction between the host genotype and pathogen. For the purpose of detailed virulence phenotyping, intermediate reactions were considered as intermediate incompatible. Next, *P. graminis* isolate UK-01 was screened for its virulence phenotype across wheat cultivars from the UK AHDB Recommended List^15^ and other wheat varieties that have historically been widely grown across the UK. Infection assays and scoring were performed as described above.

### *P. graminis* aeciospore infection assays

A total of 35 aecia were collected from *B. vulgaris* at a single location in Brandon, UK. Nine aecia were selected for infection assays and stored in damp conditions to induce release of aeciospores for up to 3 h before being applied with gentle rubbing to the leaves of the wheat varieties Vuka and Solstice and the barley variety Cassata at the seedling stage. After infection, seedlings were kept in the dark at 10 °C and high relative humidity for 24 h. Plants were then moved to a controlled environment room under long-day conditions (16 h light/8 h dark) and 19/14 °C cycle. Symptoms were recorded 14 d post infection.

### *P. graminis* ITS sequence analysis

DNA was extracted from four aecia collected on *B. vulgaris* using the CTAB method^33^ and the ITS region amplified using the primers 5ITS-SR: (5′-ATTAAAAGAATTAGAGTGCACTTT-3′) and 3ITS-SR (5′-AGATGGCAAGTGTTTTACTACT-3′). PCR products were cloned into the pGEMT-Easy vector system (Promega, USA) according to the manufacturer’s instructions. Inserts of six recombinant plasmids per amplicon were bi-directionally sequenced (GATC, Germany) and a sequence alignment of the ITS region from these aecia and 27 ITS sequences from *P. graminis* f. sp.^20,42^ was generated using MUSCLE^43^. Phylogenetic analysis was performed in MEGA 7.0^37^ using a neighbor-joining approach with bootstrap values determined from 1,000 replicates.

### Scanning electron microscopy

Samples were mounted on aluminium stubs using Tissue Tek^R^ (BDH Laboratory Supplies, Poole, England). The stubs were then immediately plunged into liquid nitrogen slush at approximately − 210 °C to cryopreserved the material. The samples were transferred onto the cryostage of an ALTO 2500 cryotransfer system (Gatan, Oxford, England) attached to a Zeiss Supra 55 VP FEG scanning electron microscope (Zeiss SMT, Germany) or the same type of cryo-system on an FEI Nova NanoSEM 450 (FEI, Eindhoven, The Netherlands). Sublimation of surface frost was performed at − 95 °C for ~ 3 min before the samples were sputter coated with platinum for 2 min at 10 mA, at colder than − 110 °C. After sputter-coating, the samples were moved onto the cryo-stage in the main chamber of the microscope, held at − 125 °C. The samples were imaged at 3 kV and digital TIFF files were stored.

### Probabilistic model of infection risk

We modelled leaf infection risk in response to microclimate using a probabilistic model^44^ parameterized for stem rust^19^. We modelled specifically germination of urediniospores on the wheat surface and subsequent penetration through stomata, as these stages are strongly constrained by free water availability^14,19,27^, in common with other rust fungi. Therefore, results provide estimates only of infection risk, not of full development of potential epidemics. The reported cardinal temperatures for spore germination, germling growth and appressorium formation are *T*_min_ = 2 °C, *T*_opt_ = 15–24 °C, and *T*_max = _30 °C^19^. The reported cardinal temperatures for penetration are *T*_min = _15 °C, *T*_opt_ = 29 °C, and *T*_max_ = 35 °C. Hence, the temperature range for penetration is considerably higher than that for germination and appressorium formation. In addition, high light availability is required for the penetration stage, reported as illumination of >10,000 lux, or approximately that received in the shade under a clear sky at noon. This reflects the pathogen likely germinating following dewfall overnight and then infecting in the morning as temperatures rise, stomata open, and dew slowly dries^19^.

A beta function was used to estimate relative rates of germination and penetration based on cardinal temperatures^44,45^, modified for germination to account for the wide optimal temperature range^46^. We modelled the transition of spores to appressoria, and appressoria to penetrations, as survival processes following a Weibull distribution^44^. In the absence of appropriate empirical data, we estimated the Weibull parameters from qualitative descriptions of the time taken for germination and penetration to occur^19^. At optimal temperatures, the Weibull processes gave near-completion of appressorium formation after 8 h and penetration in a further 3 h. The hazard functions were multiplied by the temperature-dependent rates to reduce germination and penetration rates at sub-optimal temperatures, with zero activity outside of the cardinal temperatures (Supplementary Fig. 5). Both processes occur only when leaves are wet and germinated spores die if leaves dry out.

In the UK, wheat is planted mainly in the east of England and winter wheat accounts for nearly all the wheat grown. Winter wheat is planted between September and November, tillering occurs over winter, stem elongation in spring, flowering in June, grain filling in July, and collecting in August–September. The warm temperatures required for penetration strongly suggest that the disease is most likely to strike in the summer. We obtained historical weather estimates for the summer months (June, July, and August) in the major wheat-growing regions of the United Kingdom (a rectangular grid covering 1.97°W to 1.97°E, 50.0°N to 55.0°N) from the beginning of 1990 to the end of 2016, at 3 h intervals and 0.5625° spatial resolution, from the Japanese 55-year Reanalysis (JRA55)^26^. Data were downloaded from the Research Data Archive at the National Center for Atmospheric Research, Computational and Information Systems Laboratory^47^. Weather variables required for modelling were canopy temperature (^o^C), canopy surface water content (kg m^−2^), solar irradiance (W m^−2^) and cloud cover fraction. The 3 h observations were linearly interpolated to give hourly estimates for modeling.

We assumed a constant number of spores available for germination in each hour and that germination and penetration could take place only if canopy surface water content was greater than zero. The total relative number of appressoria formed in an hour was the sum of appressoria formed by all germinating cohorts. These appressoria were then able to penetrate if moisture and light conditions allowed. JRA55 irradiance estimates were converted to estimates of illuminance (lux) using a rule-of-thumb factor of 126.6, which suggested that sufficient sunlight for penetration was available between the hours of 0800 and 2100 h. The relative number of penetrations in an hour was the sum of all penetrating cohorts and was taken as an indicator of relative infection risk.

Although 3 h projections are available for air temperature from the CMIP-5 (Coupled Model Intercomparison Project) family of Global Circulation Models (GCM)^48^, other products such as relative humidity (which can be used as an indicator of leaf wetness) are available only at coarser temporal resolutions from current repositories. Therefore, driving our germination and infection model with future projections was not possible without bespoke GCM runs. Instead, we inspected random realizations of CMIP-5 projections at daily temporal resolution provided by the Marksim weather generator^49^. We obtained 99 ensemble averages for 17 CMIP-5 GCMs for the years 2020 and 2050 under the RCP4.5 representative concentration pathway scenario for a point near Cambridge, UK, which lies near the center of the wheat-growing region of the UK, and compared temperature and precipitation estimates or the summer months for these time points.

### Code availability

All custom computer code is available at https://github.com/vbuens/Field_Pathogenomics.

### Data availability

The raw sequence data and ITS sequence data that support the findings of this study have been deposited in the European Nucleotide Archive (ENA; PRJEB22223) and Genbank (MF684370-3), respectively.

## Electronic supplementary material

Supplementary Information

Description of Additional Supplementary Files

Supplementary Data 1
