# Supplementary material for: Potential for re-emergence of wheat stem rust in the United Kingdom
Source: Commun Biol. 2018 Feb 8;1:13. doi: 10.1038/s42003-018-0013-y (PMC6053080; doi:10.1038/s42003-018-0013-y)
Supplement: Supplementary file 1 — Supplementary Information [file 42003_2018_13_MOESM1_ESM.pdf]

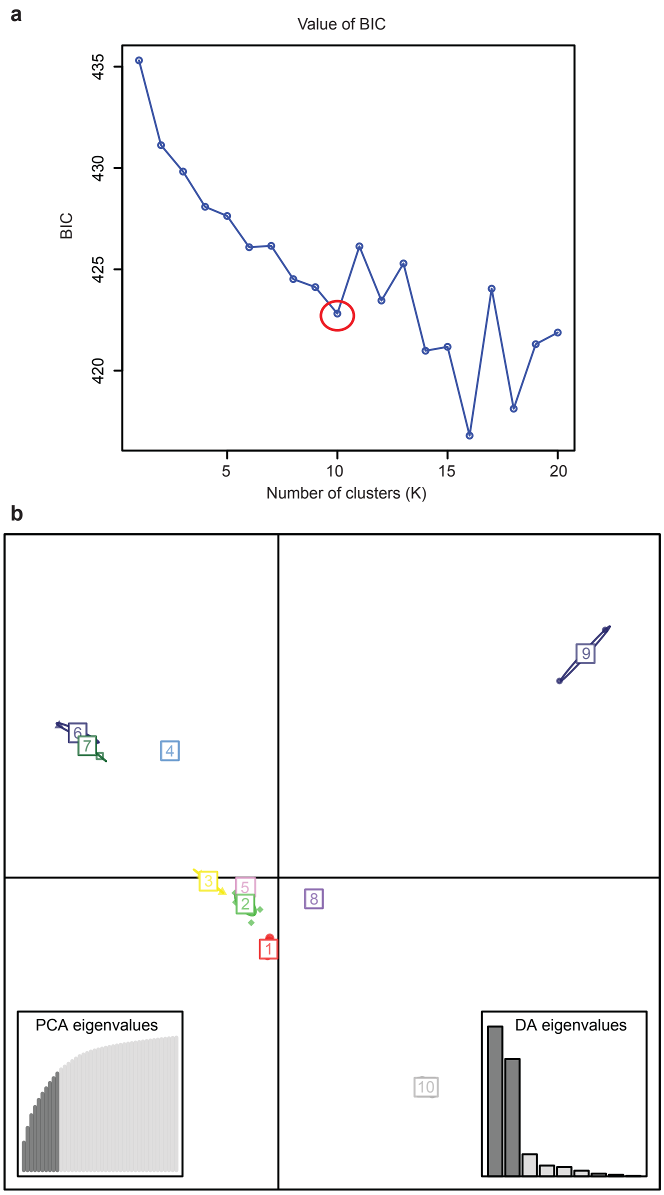

Supplementary Figure 1. Multivariate discriminant analysis of principal components (DAPC) supports the assignment of the 43 *P. graminis* f. sp. *tritici* isolates into ten genetic groups. a, The optimal predicted number of population clusters K for the dataset is ten. The Bayesian information criterion (BIC; Y-axis) was plotted for K = 1–20, and the clear 'elbow' at K = 10 indicates the optimal number of populations. **b,** Scatterplot using the first two principal components (Y-axis and X-axis, respectively) of DAPC analysis of 306,960 biallelic synonymous single nucleotide polymorphism (SNP) sites. Each symbol represents a single *P. graminis* f. sp. *tritici* isolate, which is coloured according to assignment to one of ten population clusters.

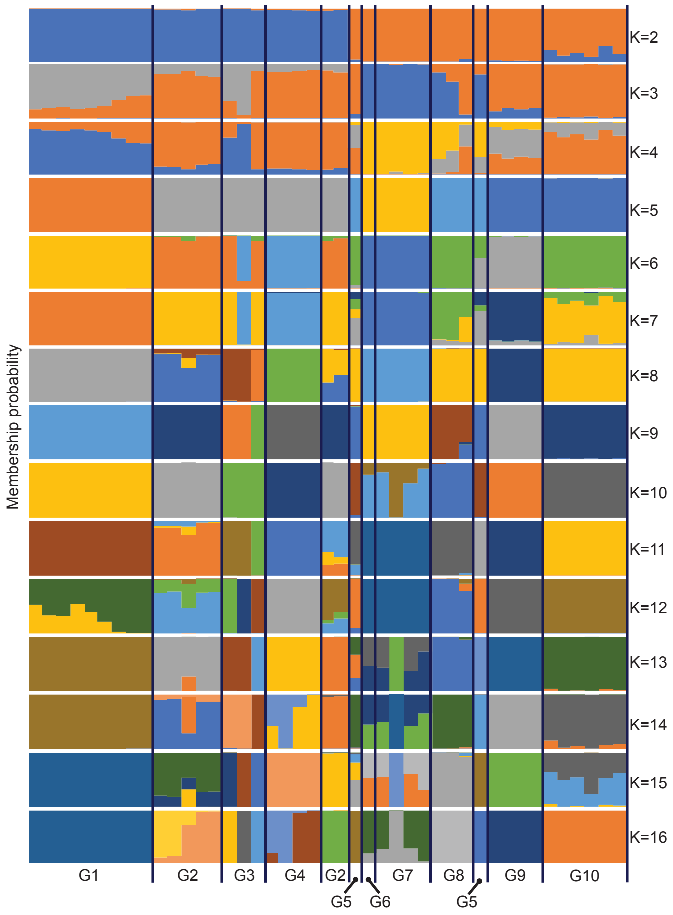

Supplementary Figure 2. Multivariate discriminant analysis of principal components (DAPC) identifies ten genetic groups in the *P. graminis* f. sp. *tritici* population. Cluster membership probabilities for individuals following DAPC analysis of 306,960 biallelic synonymous single nucleotide polymorphism (SNP) sites. Analysis was carried out for K = 2–16. Each bar represents membership fractions per individual.

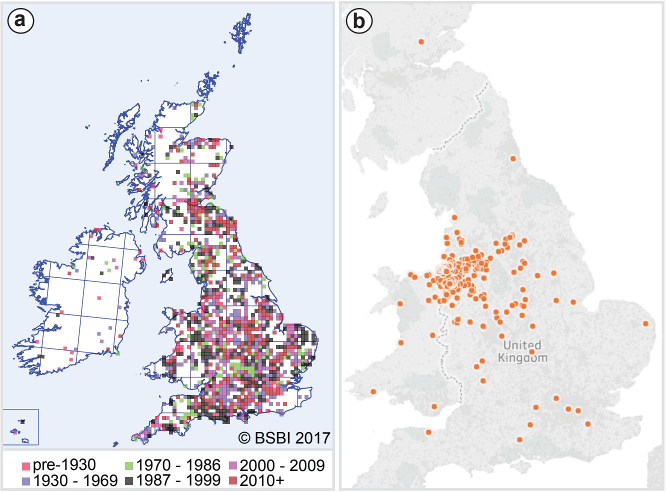

Supplementary Figure 3. *Berberis vulgaris* is widely planted across the UK and is increasing in prevalence through the conservation programme for the barberry carpet moth. a, Map illustrating the distribution of *B. vulgaris* across the UK as recorded by the Botanical Society of Britain & Ireland. **b,** Locations reported for some of the more than 1000 *B. vulgaris* plants that have been distributed to the general public by Chester Zoo to encourage re-plantation in a habitat conservation programme for the barberry carpet moth, *Pareulype berberata*.

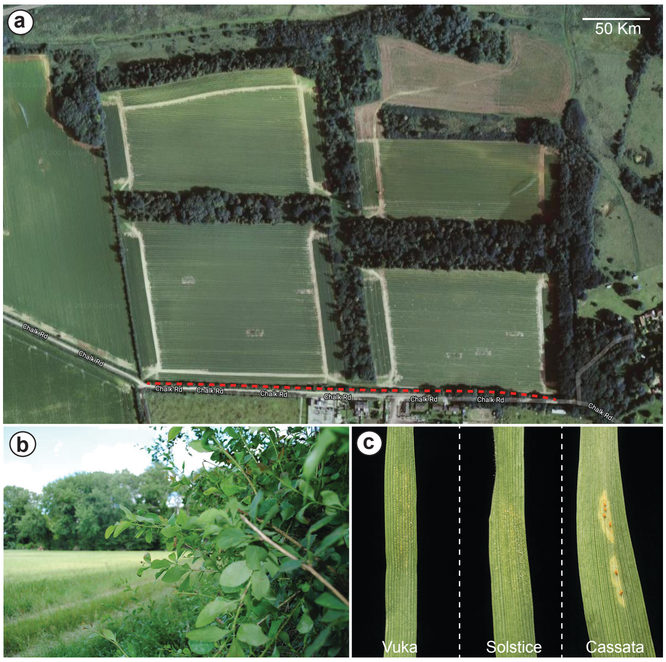

Supplementary Figure 4. Location of *Berberis vulgaris* plants identified as hosting *P. graminis* in 2017. a-b, The hedgerow in the east of England that was intermixed with barberry (red dotted line) and within a meter of a barley field. **c,** Aeciospores from the *P. graminis* isolates identified on *B. vulgaris* did not induce symptoms on the two wheat varieties tested (Vuka and Solstice), but were able to infect the selected barley variety (Cassata).

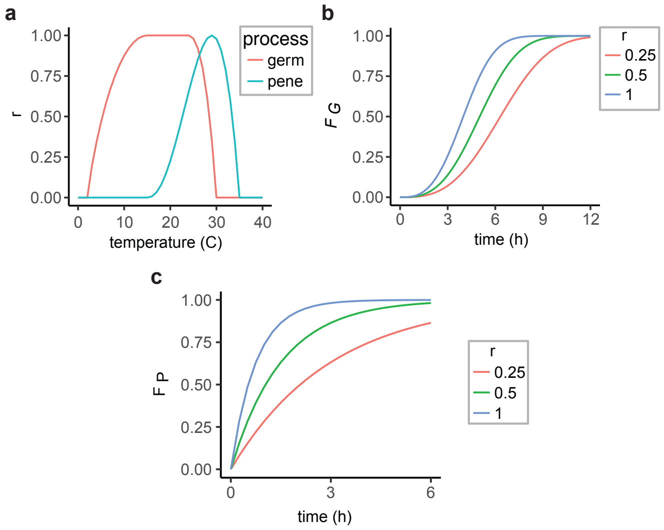

Supplementary Figure 5. Temperature response functions and cumulative distributions for germination, appressorium formation and penetration. a, Temperature response functions for germination and penetration. Relative rate (r) by temperature was estimated using a beta function parameterized by cardinal temperatures reported in the literature. **b,** Cumulative distribution function for germination and appressorium formation over time. The fraction of spores having formed appressoria (FG) over time was estimated from a Weibull function parameterized from descriptions in the literature, with shape parameter 3 and scale parameter 4.5. The Weibull hazard function was multiplied by the temperature-dependent rate, such that the maximum rate occurred at optimum temperature (blue line), and declined at suboptimal temperatures (green and red lines). **c,** Cumulative distribution function for penetration of appressoria over time. The fraction of appressoria that penetrate stomata (FP) over time was estimated from a Weibull function parameterized from descriptions in the literature (shape = 1, scale = 0.75). The Weibull hazard function was multiplied by the temperature-dependent rate, such that the maximum rate occurred at optimum temperature (blue line), and declined at suboptimal temperatures (green and red lines).

Supplementary Table 1. *P. graminis* isolates used in this study.

ID	Original isolate ID	Date collected	Country	Host	Host Variety	Genetic Group	Type of data
DK-01	DK184a_13	26/08/13	Denmark	Wheat	Kolbe	G10	RNA-seq
DK-02	DK186a_13	26/08/13	Denmark	Wheat	Borre	G10	RNA-seq
SE-01	SE373a2_14	30/07/14	Sweden	Wheat	Julius	G10	RNA-seq
CZ-01	CZ6	17/05/10	Czech Republic	Wheat	Samanta	G1	Genome
CZ-02	CZ10	18/06/13	Czech Republic	Wheat	Fermi	G2	Genome
CZ-03	CZ16	10/06/13	Czech Republic	Wheat	Akteur	G2	Genome
ET-01	SR3	16/05/14	Ethiopia	Wheat	Kingbird	G2	RNA-seq
ET-02	SR4	16/05/14	Ethiopia	Wheat	Kingbird	G2	RNA-seq
ET-03	SR15-4	23/11/15	Ethiopia	Durum Wheat	-	G10	RNA-seq
ET-04	SR15-5	23/11/15	Ethiopia	Durum Wheat	-	G10	RNA-seq
IR-01	IR SR11	02/06/15	Iran	Bread wheat	An elite line	G5	Genome
IR-02	IR SR16	01/07/15	Iran	Bread wheat	Tajan	G7	Genome
IR-03	IR SR5	16/06/15	Iran	Bread wheat	an F experiment genotype	G7	Genome
IR-04	IR SR7	20/06/15	Iran	Bread wheat	an experimental genotype	G7	Genome
IR-05	IR SR9	13/04/09	Iran	Bread wheat	-	G7	Genome
IR-06	IR SR10	02/06/15	Iran	Bread wheat	Morvarid	G6	Genome
IS-01	IS4 (2017)	1997	Israel	Bread Wheat	Dariel	G5	Genome
IS-02	IS13 (2070)	2005	Israel	Wild emmer *T. dicoccoides*	-	G8	Genome
IS-03	IS14 (2072)	2006	Israel	Bread Wheat	land race from Lebanon	G8	Genome
M-01	RKQ	1988, 1989	Mexico	Bread Wheat	-	G4	Genome
M-02	RTQ	1988, 1989	Mexico	Bread Wheat	-	G4	Genome
H-01	Pgt-H3	23/07/2002	Hungary	Wheat	-	G3	Genome
PK-01	NARC 15-15 SP	May, 2015	Pakistan	Wheat	Old wheat land race	G2	Genome
PK-02	NARC 15-36	May, 2015	Pakistan	Wheat	Old wheat land race	G2	Genome
PK-03	NARC 15-67	May, 2015	Pakistan	Wheat	Old wheat land race	G2	Genome
PK-04	15 SBPK 07 SP	20/03/15	Pakistan	Wheat	Commer cial wheat cultivar	G8	Genome
SA-01	UVPgt50	1981	South Africa	Wheat	SST66	G1	Genome
SA-02	UVPgt52	1984	South Africa	Wheat	SST44	G1	Genome
SA-03	UVPgt53	1988	South Africa	Triticale	Coorong	G1	Genome
SA-04	UVPgt54	2000	South Africa	Barley	Unknown	G1	Genome
SA-05	UVPgt56	2003	South Africa	Triticale	Kiewiet	G1	Genome
SA-06	UVPgt57	2005	South Africa	Triticale	Tobie	G1	Genome
SA-07	UVPgt58	2007	South Africa	Triticale	Pan 299	G1	Genome

UR-01	UR SR1 (2047)	2011	Uruguay	Wheat	-	G4	Genome
UR-02	UR SR2 (2058)	2011	Uruguay	Wheat	-	G4	Genome
IT-01	CER-16-04	22/05/16	Sicily, Italy	Wheat	-	G9	RNA-seq
IT-02	CER-16-05	22/05/16	Sicily, Italy	Wheat	-	G9	RNA-seq
IT-03	CER-16-06	22/05/16	Sicily, Italy	Wheat	-	G9	RNA-seq
IT-04	CER-16-07	22/05/16	Sicily, Italy	Wheat	-	G9	RNA-seq
UK-01	PGT_UK01	July, 2013	UK	Wheat	-	G10	RNA-seq
US-01	QCCJB (isolate QCC-2)	1990-92	USA	Wheat	-	G3	Genome
SE-02	Evertsholm	11/08/08	Sweden	Oats	Ingeborg	-	Genome
SE-03	Ingeberga	06/08/08	Sweden	Oats	-	-	Genome
AU-01	21-0	1954	Australia	Wheat	-	G1	Genome
AU-02	126-6,7,11	1926	Australia	Wheat	-	G3	Genome

Supplementary Table 2. Virulence profiling of *P. graminis* f. sp. *tritici* isolate UK-01, across 57 wheat varieties that include the UK Recommended List.

Wheat varieties	Reaction Type	Susceptible (S), Resistant (R)
Recommended List (RL) & recent RL		
Cordiale	3+	S
Crusoe	3+	S
KWS Crispin	3+	S
KWS Kerrin	3+	S
KWS Santiago	3+	S
KWS Trinity	3+	S
KWS Zyatt	3+	S
Leeds	3+	S
LG Motown	3+	S
LG Sundance	3+	S
Revelation	3+	S
RGT Illustrious	3+	S
Stratosphere	3+	S
Zulu	3+	S
KWS Lili	3*	S
Myriad	2+/3+	S
Freiston	2+	Intermediate
RGT Knightsbridge	2+	Intermediate
Grafton	;1	R
Belgrade	4	S
Bennington	4	S
Dunston	4	S
Graham	4	S
KWS Basset	4	S
KWS Silverstone	4	S
KWS Siskin	4	S
LG Bletchley	4	S
Reaper	4	S
Savello	4	S
Shabras	4	S
Spyder	4	S
Sterna	4	S
Viscount	4	S
Costello	2	Intermediate
Dickens	2	Intermediate
Reflection	2	Intermediate
Skyfall	2	Intermediate
Hardwicke	1	R
KWS Barrel	1	R

Moulton	1	R
JB Diego	0	R
RGT Westminster	0	R
Tuxedo	0	R
Older wheat varieties		
Armada	4	S
Avalon	4	S
Buster	4	S
Consort	2+	Intermediate
Glasgow	4	S
KWS Sterling	4	S
Maris Ranger	2+	Intermediate
Maris Fundin	0	R
Maris Halberd	4	S
Maris Huntsman	2	Intermediate
Robigus	3+	S
Soissons	1	R
Stigg	1	R
Warrior	1	R
