# Supplementary material for: Potential for re-emergence of wheat stem rust in the United Kingdom
Source: Commun Biol. 2018 Feb 8;1:13. doi: 10.1038/s42003-018-0013-y (PMC6053080; doi:10.1038/s42003-018-0013-y)
Supplement: Supplementary file 2 — Description of Additional Supplementary Files [file 42003_2018_13_MOESM2_ESM.docx]

**Description of Additional Supplementary Files**

File Name: Supplementary Data 1

Description: Quantity of reads that aligned to the P. graminis f. sp. tritici (Pgt) reference genome.
